# Do the Rich Get Richer? An Empirical Analysis of the Bitcoin Transaction Network

**DOI:** 10.1371/journal.pone.0086197

**Published:** 2014-02-05

**Authors:** Dániel Kondor, Márton Pósfai, István Csabai, Gábor Vattay

**Affiliations:** 1 Department of Physics of Complex Systems, Eötvös Loránd University, Budapest, Hungary; 2 Department of Theoretical Physics, Budapest University of Technology and Economics, Budapest, Hungary; University of Maribor, Slovenia

## Abstract

The possibility to analyze everyday monetary transactions is limited by the scarcity of available data, as this kind of information is usually considered highly sensitive. Present econophysics models are usually employed on presumed random networks of interacting agents, and only some macroscopic properties (e.g. the resulting wealth distribution) are compared to real-world data. In this paper, we analyze Bitcoin, which is a novel digital currency system, where the complete list of transactions is publicly available. Using this dataset, we reconstruct the network of transactions and extract the time and amount of each payment. We analyze the structure of the transaction network by measuring network characteristics over time, such as the degree distribution, degree correlations and clustering. We find that linear preferential attachment drives the growth of the network. We also study the dynamics taking place on the transaction network, i.e. the flow of money. We measure temporal patterns and the wealth accumulation. Investigating the microscopic statistics of money movement, we find that sublinear preferential attachment governs the evolution of the wealth distribution. We report a scaling law between the degree and wealth associated to individual nodes.

## Introduction

In the past two decades, network science has successfully contributed to many diverse scientific fields. Indeed, many complex systems can be represented as networks, ranging from biochemical systems, through the Internet and the World Wide Web, to various social systems [1]–[7]. Economics also made use of the concepts of network science, gaining additional insight to the more traditional approach [8]–[13]. Although a large volume of financial data is available for research, information about the everyday transactions of individuals is usually considered sensitive and is kept private. In this paper, we analyze Bitcoin, a novel currency system, where the complete list of transactions is accessible. We believe that this is the first opportunity to investigate the movement of money in such detail.

Bitcoin is a decentralized digital cash system, there is no single overseeing authority [14]. The system operates as an online peer-to-peer network, anyone can join by installing a client application and connecting it to the network. The unit of the currency is one bitcoin (abbreviated as BTC), and the smallest transferable amount is 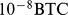. Instead of having a bank account maintained by a central authority, each user has a Bitcoin address, that consists of a pair of public and private keys. Existing bitcoins are associated to the public key of their owner, and outgoing payments have to be signed by the owner using his private key. To maintain privacy, a single user may use multiple addresses. Each participating node stores the complete list of previous transactions. Every new payment is announced on the network, and the payment is validated by checking consistency with the entire transaction history. To avoid fraud, it is necessary that the participants agree on a single valid transaction history. This process is designed to be computationally difficult, so an attacker can only hijack the system if he possesses the majority of the computational power of participating parties. Therefore the system is more secure if more resources are devoted to the validation process. To provide incentive, new bitcoins are created periodically and distributed among the nodes participating in these computations. Another way to obtain bitcoins is to purchase them from someone who already has bitcoins using traditional currency; the price of bitcoins is completely determined by the market.

**Figure 1:**
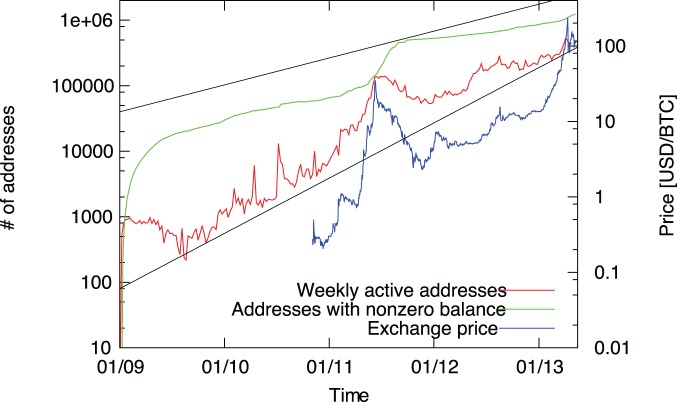
The growth of the Bitcoin network. Number of addresses with nonzero balance (green), addresses in participating in at least one transaction in one week intervals (red) and the exchange price of bitcoins in US dollars according to MtGox, the largest Bitcoin exchange site (blue). The black lines are exponential functions bounding the growth of the network size; the characteristic times are 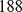 and 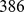 days.

The Bitcoin system was proposed in 2008 by Satoshi Nakamoto, and the system went online in January 2009 [14]–[17]. For over a year, it was only used by a few enthusiasts, and bitcoins did not have any real-world value. A trading website called MtGox was started in 2010, making the exchange of bitcoins and conventional money significantly easier. More people and services joined the system, resulting a steadily growing exchange rate. Starting from 2011, appearances in the mainstream media drew wider public attention, which led to skyrocketing prices accompanied by large fluctuations (see Fig. 1). Since the inception of Bitcoin over 17 million transactions took place, and currently the market value of all bitcoins in circulation exceeds 1 billion dollars. See the Methods section for more details of the system and the data used in our analysis.

We download the complete list of transactions, and reconstruct the transaction network: each node represents a Bitcoin address, and we draw a directed link between two nodes if there was at least one transaction between the corresponding addresses. In addition to the topology, we also obtain the time and amount of every payment. Therefore, we are able to analyze both the evolution of the network and the dynamical process taking place on it, i.e. the flow and accumulation of bitcoins. To characterize the underlying network, we investigate the evolution of basic network characteristics over time, such as the degree distribution, degree correlations and clustering. Concerning the dynamics, we measure the wealth statistics and the temporal patterns of transactions. To explain the observed degree and wealth distribution, we measure the microscopic growth statistics of the system. We provide evidence that preferential attachment is an important factor shaping these distributions. Preferential attachment is often referred to as the “rich get richer” scheme, meaning that hubs grow faster than low-degree nodes. In the case of Bitcoin, this is more than an analogy: we find that the wealth of already rich nodes increases faster than the wealth of nodes with low balance; furthermore, we find positive correlation between the wealth and the degree of a node.

## Results

### Evolution of the Transaction Network

Bitcoin is an evolving network: new nodes are added by creating new Bitcoin addresses, and links are created if there is a transaction between two previously unconnected addresses. The number of nodes steadily grows over time with some fluctuations; especially noticeable is the large peak which coincides with the first boom in the exchange rate in 2011 (Fig. 1). After five years Bitcoin now has 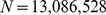 nodes and 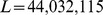 links. To study the evolution of the network we measure the change of network characteristics in function of time. We identify two distinct phases of growth: (i) The *initial phase* lasted until the fall of 2010, in this period the system had low activity and was mostly used as an experiment. The network measures are characterized by large fluctuations. (ii) After the initial phase the Bitcoin started to function as a real currency, bitcoins gained real value. The network measures converged to their typical value by mid-2011 and they did not change significantly afterwards. We call this period the *trading phase*.

**Figure 2:**
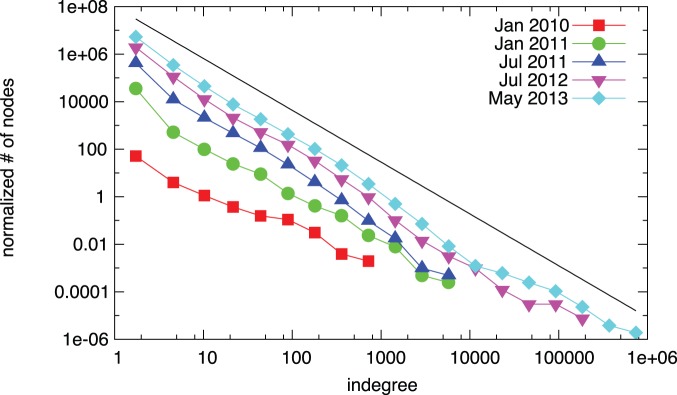
Evolution of the indegree distribution. Since the beginning of 2011, the shape of the distribution does not change significantly. The black line shows a fitted power-law for the final network; the exponent is 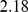. The data is log-binned for ease of visual inspection, the power-law is fitted on the original data [18].

**Figure 3:**
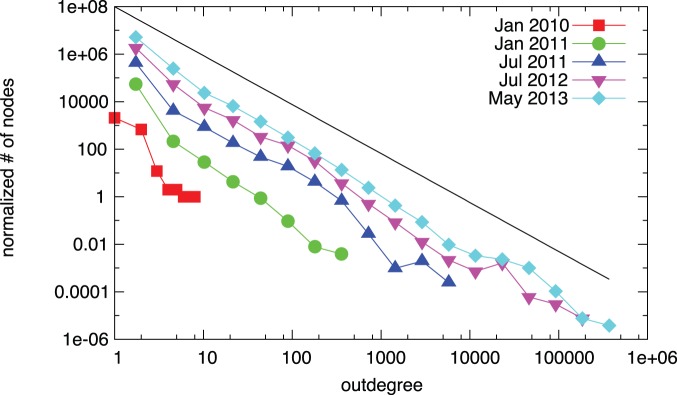
Evolution of the outdegree distribution. The black line shows a fitted power-law for the final network; the exponent is 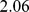. The data is log-binned for ease of visual inspection, the power-law is fitted on the original data [18].

We first measure the degree distribution of the network. We find that both the in- and the outdegree distributions are highly heterogeneous, and they can be modeled with power-laws [18]. Figures 2 and 3 show the distribution of indegrees and outdegrees at different points of time during the evolution of the Bitcoin network. In the initial phase the number of nodes is low, and thus fitting the data is prone to large error. In the trading phase, the exponents of the distributions do not change significantly, and they are approximated by power-laws 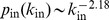 and 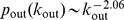.

**Figure 4:**
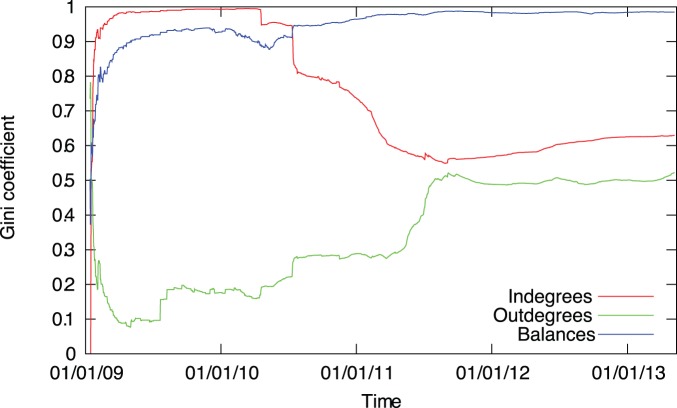
Evolution of the Gini coefficient of the degree and the balance distributions. We observe the distinct initial phase lasting until mid-2011. The trading phase is characterized by approximately constant coefficients.

To further characterize the evolution of the degree distributions we calculate the corresponding Gini coefficients in function of time (Fig. 4). The Gini coefficient is mainly used in economics to characterize the inequality present in the distribution of wealth, but it can be used to measure the heterogeneity of any empirical distribution. In general, the Gini coefficient is defined as.
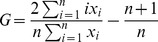
(1)where 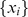 is a sample of size 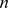, and 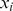 are monotonically ordered, i.e. 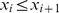. 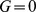 indicates perfect equality, i.e. every node has the same wealth; and 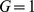 corresponds to complete inequality, i.e. the complete wealth in the system is owned by a single individual. For example, in the case of pure power-law distribution with 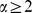 exponent, the Gini coefficient is 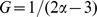 [19]. This shows the fact that smaller 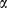 exponents yield more heterogeneous wealth distributions.

In the Bitcoin network we find that in the initial phase the Gini coefficient of the indegree distribution is close to 1 and for the outdegree distribution it is much lower. We speculate that in this phase a few users collected bitcoins, and without the possibility to trade, they stored them on a single address. In the second phase the coefficients quickly converge to 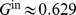 and 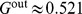, indicating that normal trade is characterized by both highly heterogeneous in- and outdegree distributions.

To characterize the degree correlations we measure the Pearson correlation coefficient of the out- and indegrees of connected node pairs:
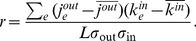
(2)


Here 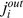 is the outdegree of the node at the *beginning* of link 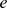, and 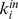 is the indegree of the node at the *end* of link 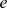. The summation 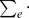 runs over all links, 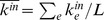 and 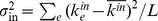. We calculate 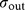 and 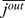 similarly.

**Figure 5:**
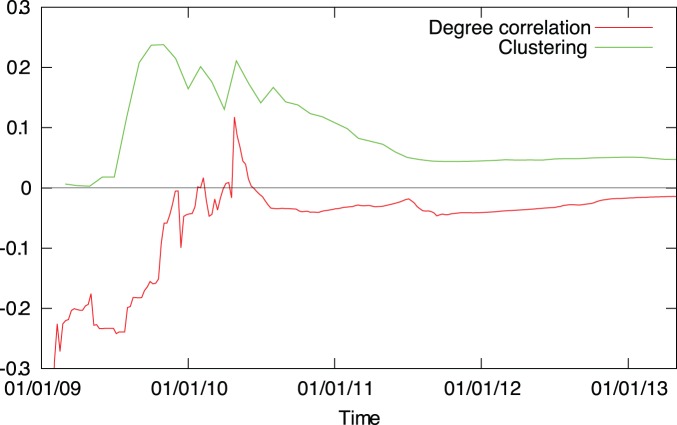
Evolution of the clustering coefficient and the out-in degree correlation coefficient. After the initial phase, both measures reach a stationary value.

**Figure 6:**
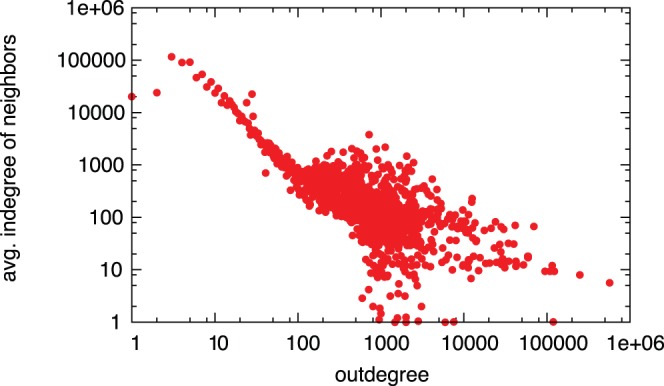
The average indegree of neighbors in the function of the outdegree 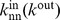. In networks without degree correlations, the degree of connected nodes do not depend on each other, therefore for such networks we expect that 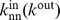 is constant. In the case of the Bitcoin network, we observe a clear disassortative behavior: 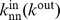 is a decreasing function, indicating that nodes with high outdegree tend to connect to nodes with low indegree.

We find that the correlation coefficient is negative, except for only a brief period in the initial phase. After mid-2010, the degree correlation coefficient stays between 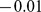 and 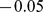, reaching a value of 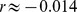 by 2013, suggesting that the network is disassortative (Fig. 5). However, small values of 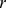 are hard to interpret: it was shown that for large purely scale-free networks 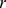 vanishes as the network size increases [20]. Therefore we compute the average nearest neighbor degree function 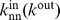 for the final network; 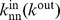 measures the average indegree of the neighbors of nodes with outdegree 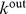. We find clear disassortative behavior (Fig. 6).

We also measure the average clustering coefficient.
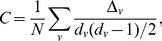
(3)which measures the density of triangles in the network. Here the sum 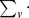 runs over all nodes, and 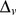 is the number of triangles containing node 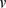. To calculate 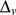 we ignored the directionality of the links; 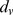 is the degree of node 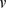 in the undirected network.

In the initial phase 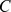 is high, fluctuating around 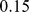 (see Fig. 5), possibly a result of transactions taking place between addresses belonging to a few enthusiasts trying out the Bitcoin system by moving money between their own addresses. In the trading phase, the clustering coefficient reaches a stationary value around 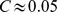, which is still higher than the clustering coefficient for random networks with the same degree sequence (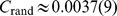).

To explain the observed broad degree distribution, we turn to the microscopic statistics of link formation. Most real complex networks exhibit distributions that can be approximated by power-laws. Preferential attachment was introduced as a possible mechanism to explain the prevalence of this property [21]. Indeed, direct measurements confirmed that preferential attachment governs the evolution of many real systems, e.g. scientific citation networks [22]–[24], collaboration networks [25], social networks [26], [27] or language use [28]. In its original form, preferential attachment describes the process when the probability of forming a new link is proportional to the degree of the target node [29]. In the past decade, several generalizations and modifications of the original model were proposed, aiming to reproduce further structural characteristics of real systems [30]–[33]. Here, we investigate the nonlinear preferential attachment model [30], where the probability that a new link connects to node 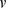 is.
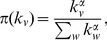
(4)where 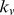 is the indegree of node 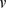, and 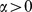. The probability that the new link connects to *any* node with degree 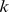 is 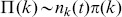, where 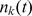 is the number of nodes with 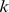 degree at the time of the link formation. We cannot test directly our assumption, because 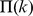 changes over time. To proceed we transform 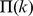 to a uniform distribution by calculating the rank function 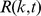 for each new link given 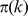 and 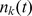:



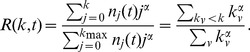
(5)If Eq. 4 holds, 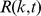 is uniformly distributed in the interval 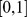, independently of 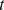. Therefore, if we plot the cumulative distribution function, we get a straight line for the correct exponent 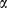. To determine the best exponent, we compare the empirical distribution of the 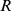 values to the uniform distribution for different exponents by computing the Kolmogorov-Smirnoff distance between the two distributions.

**Figure 7:**
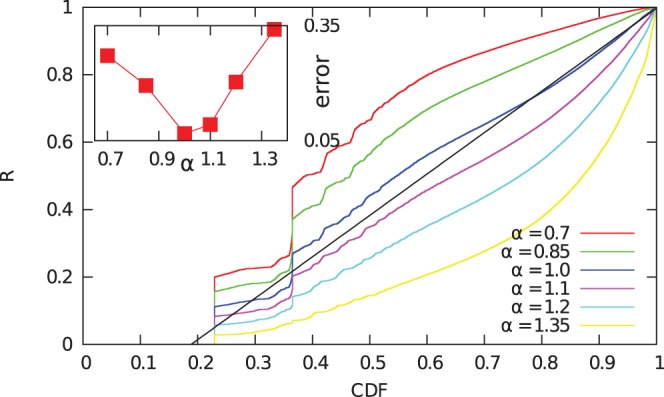
Rank function for new link creation. The cumulative distribution function of the 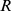 values (see Eq. 5) for exponents 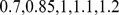 and 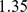. The inset shows the Kolmogorov-Smirnoff error for these exponents.

Evaluating our method for indegree distribution of the Bitcoin network, we find good correspondence between the empirical data and the presumed conditional probability function; the exponent giving the best fit is 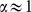 (Fig. 7). This shows that the overall growth statistics agree well with the preferential attachment process. Of course, preferential attachment itself cannot explain the disassortative degree correlations and the high clustering observed in the network. We argue that preferential attachment is a key factor shaping the degree distribution, however, more detailed investigation of the growth process is necessary to explain the higher order correlations.

### Dynamics of Transactions

In the this section, we analyze the detailed dynamics of money flow on the transaction network. The increasing availability of digital traces of human behavior revealed that various human activities, e.g. mobility patterns, phone calls or email communication, are often characterized by heterogeneity [34]–[37]. Here we show that the handling of money is not an exception: we find heterogeneity in both balance distribution and temporal patterns. We also investigate the microscopic statistics of transactions.

The state of node 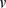 at time 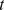 is given by the balance of the corresponding address 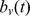, i.e. the number of bitcoins associated to node 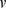. The transactions are directly available, and we can infer the balance of each node based on the transaction list. Note that the overall quantity of bitcoins increases over time: Bitcoin rewards users devoting computational power to sustain the system.

**Figure 8:**
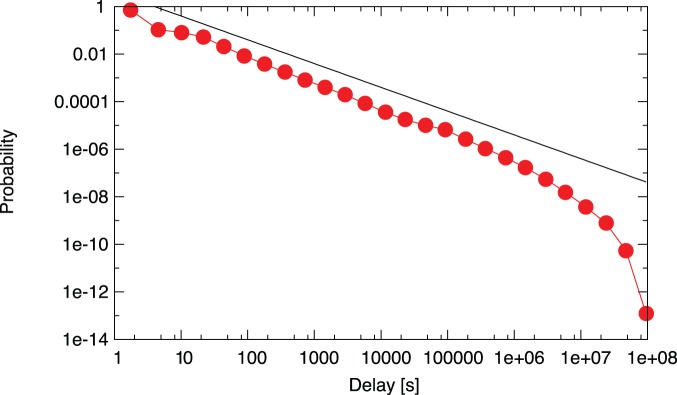
Distribution of time delay between transactions initiated from a single Bitcoin address. We observe a power-law distribution close to the widely observed 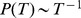, the exponential cutoff corresponds to the finite lifetime of the Bitcoin system.

We first investigate the temporal patterns of the system by measuring the distribution of inactivity times 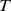. The inactivity time is defined as the time elapsed between two consecutive outgoing transactions from a node. We find a broad distribution that can be approximated by the power-law 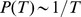 (Fig. 8), in agreement with the behavior widely observed in various complex systems [34], [38]–[40].

**Figure 9:**
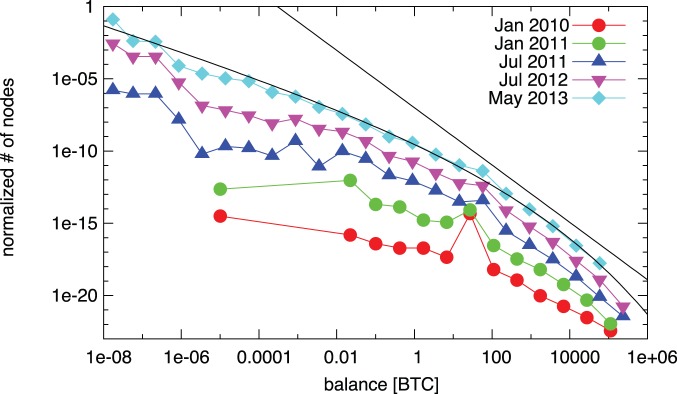
Evolution of the distribution of balances of individual Bitcoin addresses. The distributions are shifted by arbitrary factors along the vertical axis for better visibility of the separate lines. The black lines are stretched exponential and power-law fits of the last empirical distribution. The tail can be approximated by a power-law with exponent 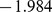, however, the rest of the fit is unsatisfactory. Therefore, we fit the distribution with a stretched exponential distribution of form 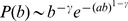. We find a better approximation of the whole distributions; the parameters are 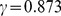 and 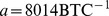.

It is well known that the wealth distribution of society is heterogeneous; the often cited –and quantitatively not precise–80–20 rule of Pareto states that the top 20% of the population controls 80% of the total wealth. In line with this, we find that the wealth distribution in the Bitcoin system is also highly heterogeneous. The proper Pareto-like statement for the Bitcoin system would be that the 6.28% of the addresses posesses the 93.72% of the total wealth. We measure the distribution of balances at different points of time, and we find a stable distribution. The tail of wealth distribution is generally modeled with a power-law [41]–[43], following this practice we find a power-law tail 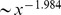 for balances 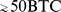 (see Fig. 9). However, visual inspection of the fit is not convincing: the scaling regime spans only the last few orders of magnitude, and fails to reproduce the majority of the distribution. Instead we find that the overall behavior is much better approximated by the stretched exponential distribution 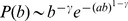, where 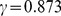 and 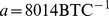.

To further investigate the evolution of the wealth distribution we measure the Gini coefficient over time. We find that the distribution is characterized by high values throughout the whole lifetime of the network, reaching a stationary value around 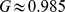 in the trading phase (see Fig. 4).

To understand the origin of this heterogeneity, we turn to the microscopic statistics of acquiring bitcoins. Similarly to the case of degree distributions, the observed heterogeneous wealth distributions are often explained by preferential attachment. Moreover, preferential attachment was proposed significantly earlier in the context of wealth distributions than complex networks [44]. In economics preferential attachment is traditionally called the Matthew effect or the “rich get richer phenomenon” [45]. It states that the growth of the wealth of each individual is proportional to the wealth of that individual. In line with this principle, several statistical models were proposed to account for the heterogeneous wealth distribution [41], [46]–[48].

**Figure 10:**
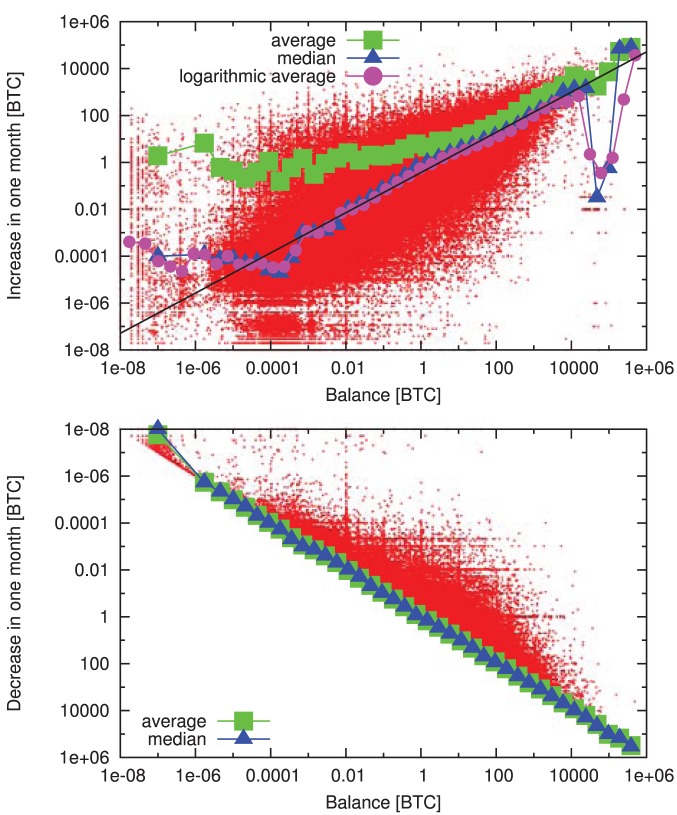
Change of balances in one month windows. Increase (top) and decrease (bottom, vertical axis is inverted) of node balances in one month windows as a function of their balance at the beginning of each month. We show the raw data (red), the average (green), median (blue) and logarithmic average (magenta). The later three are calculated for logarithmically sized bins. We find a clear positive correlation: addresses with high balance typically increase their wealth more than addresses with low balance. The median and the logarithmic average values almost coincide, which suggests multiplicative fluctuations. The median and the logarithmic average increase approximately as power-laws for several orders of magnitude. The black line is a power-law fit for the double logarithmic data; the exponent is 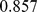.

To find evidence supporting this hypothesis, we first investigate the change of balances in fixed time windows. We calculate the difference between the balance of each address at the end and at the start of each month. We plot the differences in function of the starting balances (Fig. 10). When the balance increases, we observe a positive correlation: the average growth increases in function of the starting balance, and it is approximated by the power-law 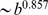. This indicates the “rich get richer” phenomenon is indeed present in the system. For decreasing balances, we find that a significant number of addresses lose all their wealth in the time frame of one month. This phenomenon is specific to Bitcoin: due to the privacy concerns of users, it is generally considered a good practice to move unspent bitcoins to a new address when carrying out a transaction [49].

**Figure 11:**
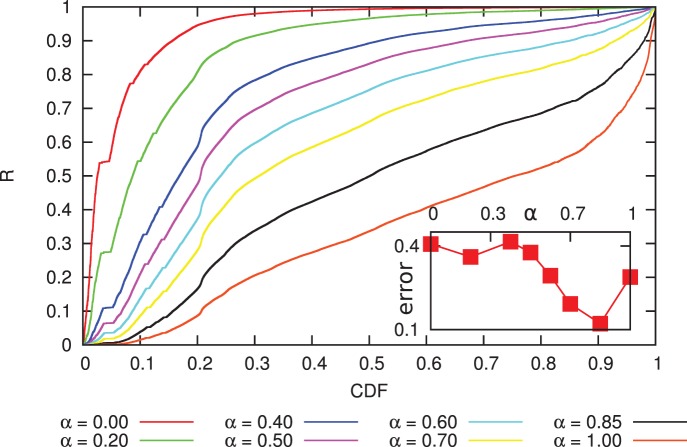
Rank function for the growth of balances. The cumulative distribution function of the 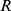 values (see Eq. 5) for exponents 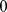, 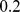, 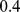, 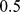, 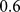, 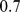, 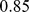 and 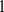. The inset shows the maximum Kolmogorov-Smirnoff error for these exponents. Here, the results are not as obvious as in the case of link creation (Fig. 7; a simple power-law form like in Eq. 4 is not sufficient to accurately model the statistics of money flow. On the other hand, the “average” behavior shows a correlation between the balance and the increase of the balance: the uncorrelated assumption (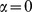) clearly gives a much worse approximate than the exponents that presume preferential attachment (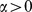).

To better quantify the preferential attachment, we carry out a similar analysis to the previous section. However, there is a technical difference: in the case of the evolution of the transaction network, for each event the degree of a node increases by exactly one. In the case of the wealth distribution there is no such constraint. To overcome this difficulty we consider the increment of a node’s balance by one unit as an event, e.g. if after a transaction 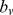 increased by 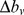, we consider it as 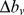 separate and simultaneous events. We only consider events when the balance associated to an address increases, i.e. the address receives a payment. We now calculate the rank function 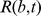 defined in Eq. 5, and plot the cumulative distribution function of the 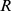 values observed throughout the whole time evolution of the Bitcoin network (Fig. 11). Visual inspection shows that no single exponent provides a satisfying result, meaning that 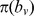 cannot be modeled by a simple power-law relationship like in Eq. 4. However, we do find that the “average” behavior is best approximated by exponents around 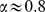, suggesting that 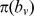 is a sublinear function. In the context of network evolution, previous theoretical work found that sublinear preferential attachment leads to a stationary stretched exponential distribution [30], in line with our observations.

**Figure 12:**
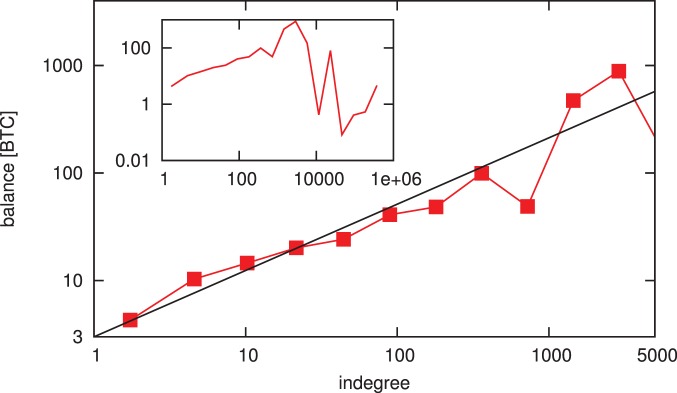
Average node balances as a function of the indegrees. We calculate the averages for logarithmically sized bins. We find strong correlation between the balance and the indegree of individual nodes. The main plot shows indegree values up to 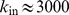, only 75 nodes (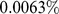) have higher indegree, the averages calculated for such small sample result in high fluctuations (see inset). We also measure both the Pearson and Spearman correlation coefficient: The Pearson correlation coefficient of the full dataset is 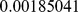, while the Spearman rank correlation coefficient is 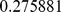. (Note that the Pearson correlation coefficient measures the linear dependence between two variables, while the Spearman coefficient evaluates monotonicity). We test the statistical significance of the correlation by randomizing the dataset 1000 times and calculating the Spearman coefficient for each randomization. We find that the average Spearman coefficient is 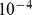 with a standard deviation of 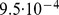, indicating that the correlation is indeed significant.

We have investigated the evolution of both the transaction network and the wealth distribution separately. However, it is clear that the two processes are not independent. To study the connection between the two, we measure the correlation between the indegree and balance associated to the individual nodes. We plot the average balance of addresses as a function of their degrees on Fig. 12. For degrees in the range of 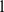
–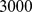 (over 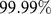 of all nodes with nonzero balance), the average balance is a monotonously increasing function of the degree, and it is approximated by the power-law 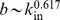, indicating that the accumulated wealth and the number of distinct transaction partners an individual has are inherently related. Similar scaling was reported by Tseng et al., who conducted an online experiment where volunteers traded on a virtual market [48].

## Methods

### The Bitcoin Network

**Figure 13:**
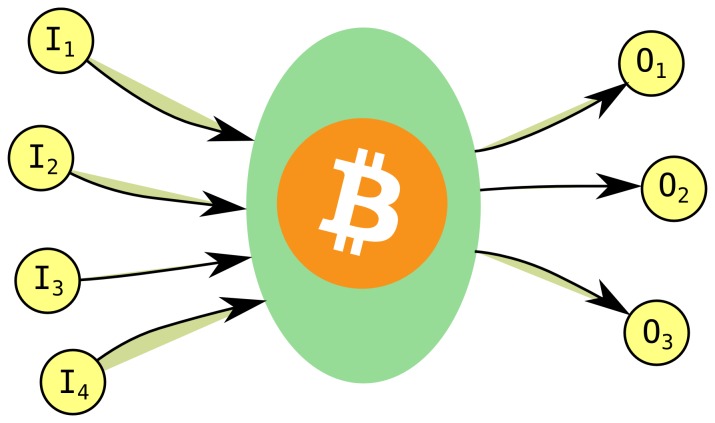
Schematic view of a Bitcoin transaction. Here we have four input (I
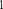
–I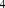) and three output (O
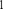
–O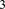) addresses. Links in our analysis are created pointing from each input to each output address.

Bitcoin is based on a peer-to-peer network of users connected through the Internet, where each node stores the list of previous transactions and validates new transactions based on a proof-of-work system. Users announce new transactions on this network, these transactions are formed into *blocks* at an approximately constant rate of one block per 10 minutes; blocks contain a varying number of transactions. These blocks form the block-chain, where each block references the previous block. Changing a previous transaction (e.g. double spending) would require the recomputation of all blocks since then, which becomes practically infeasible after a few blocks. To send or receive bitcoins, each user needs at least one address, which is a pair of private and public keys. The public key can be used for receiving bitcoins (users can send money to each other referencing the recipient’s public key), while sending bitcoins is achieved by signing the transaction with the private key. Each transaction consists of one or more *inputs* and *outputs*. In Fig. 13 we show a schematic view of a typical Bitcoin transaction. Readers interested in the technical details of the system can consult the original paper by Satoshi Nakamoto [14] or the various resources available on the Internet [50], [51].

An important aspect of Bitcoin is how new bitcoins are created, and how new users can acquire bitcoins. New bitcoins are generated when a new block is formed as a reward to the users participating in block generation. The generation of a valid new block involves solving a reverse hash problem, whose difficulty can be set in a wide range. Participating in block generation is referred to as *mining* bitcoins. The nodes in the network regulate the block generation process by adjusting the difficulty to match the processing power currently available. As interest in the Bitcoin system grew, the effort required to generate new blocks, and thus receive the newly available bitcoins, has increased over 10 million fold; most miners today use specialized hardware, requiring significant investments. Consequently, an average Bitcoin user typically acquires bitcoins by either buying them at an exchange site or receiving them as compensation for goods or services.

Due to the nature of the system, the record of all previous transactions since its beginning are publicly available to anyone participating in the Bitcoin network. From these records, one can recover the sending and receiving addresses, the sum involved and the approximate time of the transaction. Such detailed information is rarely available in financial systems, making the Bitcoin network a valuable source of empirical data involving monetary transactions. Of course, there are shortcomings: only the addresses involved in the transactions are revealed, not the users themselves. While providing complete anonymity is not among the stated goals of the Bitcoin project [52], identifying addresses belonging to the same user can be difficult [16], especially on a large scale. Each user can have an unlimited number of Bitcoin addresses, which appear as separate nodes in the transaction records. When constructing the network of users, these addresses would need to be joined to a single entity.

Another issue arises not only for Bitcoin, but for most online social datasets: It is hard to determine which observed phenomena are specific to the system, and which results are general. We do not know to what extent the group of people using the system can be considered as a representative sample of the society. In the case of Bitcoin for example, due to the perceived anonymity of the system, it is widely used for commerce of illegal items and substances [53]; these types of transactions are probably overrepresented among Bitcoin transactions. Ultimately, the validity of our results will be tested if data becomes available from other sources, and comparison becomes possible.

### Data

We installed the open-source bitcoind client and downloaded the blockchain from the peer-to-peer network on May 7th, 2013. We modified the client to extract the list of all transactions in a human-readable format. We downloaded more precise timestamps of transactions from the blockchain.info website’s archive. The data and the source code of the modified client program is available at the project’s website [54] or through the Casjobs web database interface [55], [56].

The data includes 235,000 blocks, which contain a total of 17,354,797 transactions. This dataset includes 13,086,528 addresses (i.e. addresses appearing in at least one transaction); of these, 1,616,317 addresses were active in the last month. The Bitcoin network itself does not store balances associated with addresses, these can be calculated from the sum of received and sent bitcoins for each address; preventing overspending is done by requiring that the input of a transaction corresponds to the output of a previous transaction. Using this method, we found that approximately one million addresses had nonzero balance at the time of our analysis.

## Discussion

We have preformed detailed analysis of Bitcoin, a novel digital currency system. A key difference from traditional currencies handled by banks is the open nature of the Bitcoin: each transactions is publicly announced, providing unprecedented opportunity to study monetary transactions of individuals. We have downloaded and compiled the complete list of transactions, and we have extracted the time and amount of each payment. We have studied the structure and evolution of the transaction network, and we have investigated the dynamics taking place on the network, i.e. the flow of bitcoins.

Measuring basic network characteristics in function of time, we have identified two distinct phases in the lifetime of the system: (i) When the system was new, no businesses accepted bitcoins as a form of payment, therefore Bitcoin was more of an experiment than a real currency. This initial phase is characterized by large fluctuations in network characteristics, heterogeneous indegree- and homogeneous outdegree distribution. (ii) Later Bitcoin received wider public attention, the increasing number of users attracted services, and the system started to function as a real currency. This trading phase is characterized by stable network measures, dissasortative degree correlations and power-law in- and outdegree distributions. We have measured the microscopic link formation statistics, finding that linear preferential attachment drives the growth of the network.

To study the accumulation of bitcoins we have measured the wealth distribution at different points in time. We have found that this distribution is highly heterogeneous through out the lifetime of the system, and it converges to a stable stretched exponential distribution in the trading phase. We have found that sublinear preferential attachment drives the accumulation of wealth. Investigating the correlation between the wealth distribution and network topology, we have identified a scaling relation between the degree and wealth associated to individual nodes, implying that the ability to attract new connections and to gain wealth is fundamentally related.

We believe that the data presented in this paper has great potential to be used for evaluating and refining econophysics models, as not only the bulk properties, but also the microscopic statistics can be readily tested. To this end, we make all the data used in this paper available online to the scientific community in easily accessible formats [54]–[56].
