# Correction: Do the Rich Get Richer? An Empirical Analysis of the Bitcoin Transaction Network

**DOI:** 10.1371/journal.pone.0097205

**Published:** 2014-05-02

**Authors:** The PLOS ONE Staff

In the PDF and XML versions of the article, the points within Figure 10 are blurred. Please see a higher quality version of the figure below.

**Figure 10:**
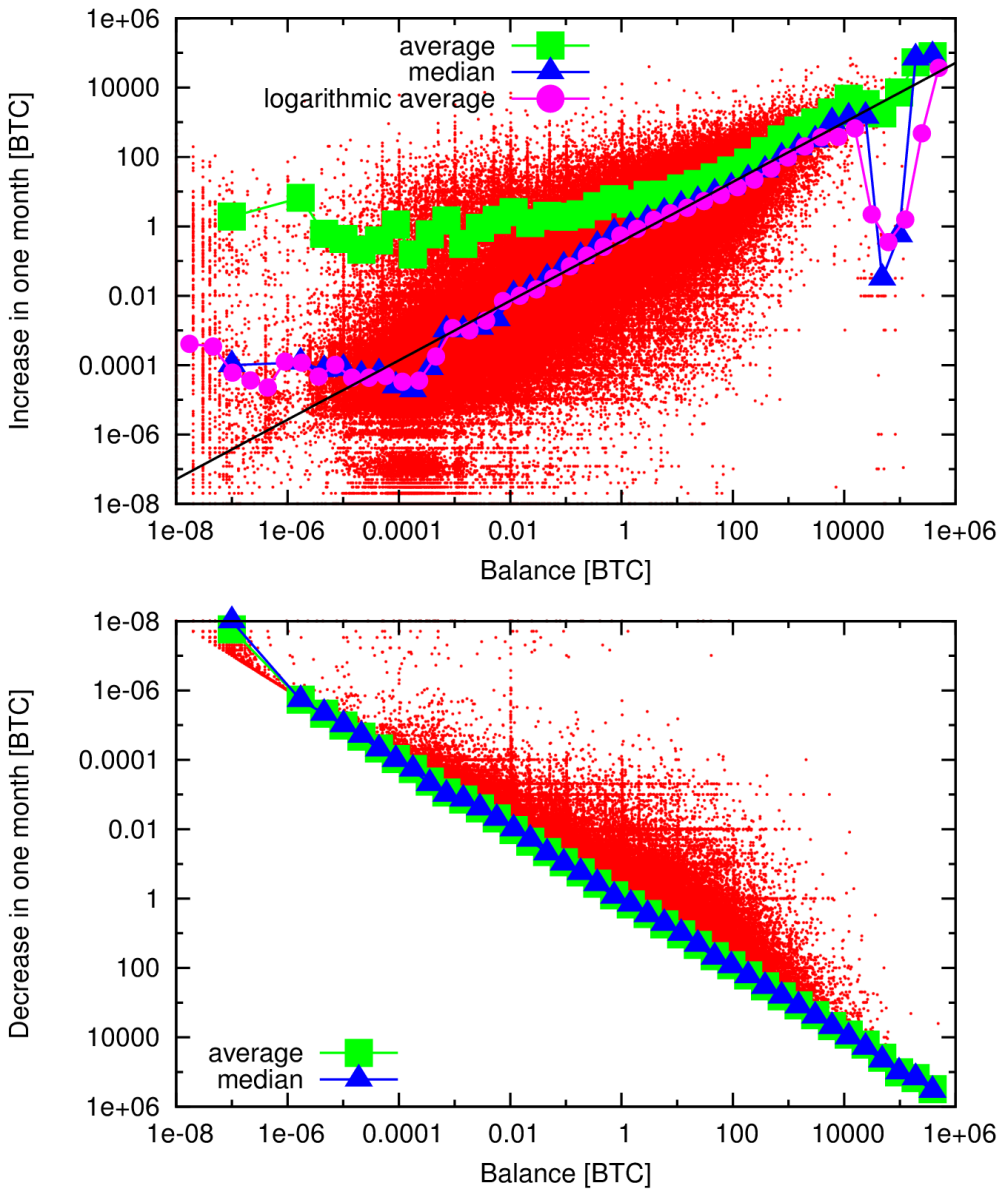
Change of balances in one month windows. Increase (top) and decrease (bottom, vertical axis is inverted) of node balances in one month windows as a function of their balance at the beginning of each month. We show the raw data (red), the average (green), median (blue) and logarithmic average (magenta). The later three are calculated for logarithmically sized bins. We find a clear positive correlation: addresses with high balance typically increase their wealth more than addresses with low balance. The median and the logarithmic average values almost coincide, which suggests multiplicative fluctuations. The median and the logarithmic average increase approximately as power-laws for several orders of magnitude. The black line is a power-law fit for the double logarithmic data; the exponent is 0.857.
